# Lipid and protein maps defining arterial layers in atherosclerotic aorta

**DOI:** 10.1016/j.dib.2015.06.005

**Published:** 2015-06-24

**Authors:** Marta Martin-Lorenzo, Benjamin Balluff, Aroa S. Maroto, Ricardo J. Carreira, Rene J.M. van Zeijl, Laura Gonzalez-Calero, Fernando de la Cuesta, Maria G Barderas, Luis F Lopez-Almodovar, Luis R Padial, Liam A. McDonnell, Fernando Vivanco, Gloria Alvarez-Llamas

**Affiliations:** a Department of Immunology, IIS-Fundacion Jimenez Diaz, UAM, REDinREN, Madrid, Spain; b Center for Proteomics and Metabolomics, Leiden University Medical Center, Leiden, The Netherlands; c Department of Vascular Physiopathology, Hospital Nacional de Paraplejicos, SESCAM, Toledo, Spain; d Department of Cardiac Surgery, Hospital Virgen de la Salud, SESCAM, Toledo, Spain; e Department of Cardiology, Hospital Virgen de la Salud, SESCAM, Toledo, Spain; f Department of Biochemistry and Molecular Biology I, Universidad Complutense, Madrid, Spain

## Abstract

Subclinical atherosclerosis cannot be predicted and novel therapeutic targets are needed. The molecular anatomy of healthy and atherosclerotic tissue is pursued to identify ongoing molecular changes in atherosclerosis development. Mass Spectrometry Imaging (MSI) accounts with the unique advantage of analyzing proteins and metabolites (lipids) while preserving their original localization; thus two dimensional maps can be obtained. Main molecular alterations were investigated in a rabbit model in response to early development of atherosclerosis. Aortic arterial layers (intima and media) and calcified regions were investigated in detail by MALDI-MSI and proteins and lipids specifically defining those areas of interest were identified. These data further complement main findings previously published in J Proteomics (M. Martin-Lorenzo et al., J. Proteomics. (In press); M. Martin-Lorenzo et al., J. Proteomics 108 (2014) 465–468.) [1,2].

Specifications table

**Table:** 

Subject area	Biology
More specific subject area	Cardiovascular disease, MSI development and application to arterial tissue
Type of data	Table and figure
How data was acquired	MALDI-MSI, FTICR
Data format	Analyzed
Experimental factors	Specific and careful tissue treatment was applied as previously published [1]
Experimental features	
Data source location	LUMC (Leiden, The Netherlands), IIS-Fundación Jiménez Díaz (Madrid, Spain)
Data accessibility	

Value of the data

• A novel unexplored ex vivo imaging approach in cardiovascular disease;
• 30 µm high spatial resolution is applied to investigate atherosclerosis tissue layers;
• This is the first time specific protein localization and alteration in response to atherosclerosis is shown by MALDI-MSI;
• TMSB4X up-regulation in atherosclerosis is firstly identified at its original location.

## 1 Data, experimental design, materials and methods

### 1.1 Data

**Fig. 1:**
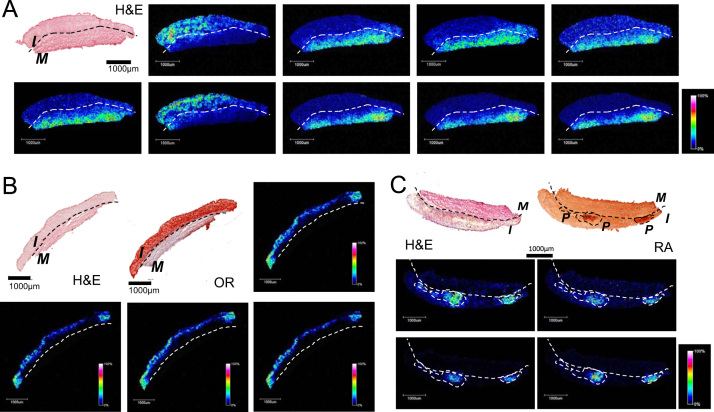
Representative MALDI-MSI images for proteins (A) and lipids (B, *C*) in rabbit aorta. Intima (I) and media (M) layers and calcified regions (*P*) in the intima are defined by specific *m*/*z* values. Characterization of samples is made according to histology: H&E, Oil-Red (OR) and Red Alizarin (RA).

Specific molecular features (*m*/*z* values) were identified by MALDI-MSI, corresponding to proteins and lipids specifically defining intima, media or calcified regions in atherosclerotic rabbit aorta (Fig. 1). *m*/*z* values with specific location, and fold change in response to atherosclerosis early development are compiled in Table 1. Tentative identification was performed and is also shown.

### 1.2 Experimental design

A rabbit model of atherosclerosis was developed as previously published [3] to investigate molecular alterations in arterial tissue in response to atherosclerosis. High-spatial-resolution MALDI-MSI was applied to comparatively analyze histologically-based arterial regions of interest from control and atherosclerotic aortas.

### 1.3 Materials and methods

The ascending aortic section of each animal was dissected, snap frozen in liquid nitrogen without any fixation and stored at −80 °C [4,5].Three different MALDI-MSI protocols were applied for the detection of proteins [2], lipids [6] and metabolites [7,8]. Public libraries of MALDI-MSI data, MSiMass list database [9] and MaTisse [10] were used to assign identity of the most significantly altered protein molecular feature using a mass tolerance of ±3 Da [11]. Lipid molecular identification was performed by using exact mass measurements, peak peaking and spatial filtering combined with Lipidsmap database using a tolerance of ≤0.005 Da, as previously published [12,13]. For comparison between control and atherosclerotic tissue, a random selection of the whole spectra sets from these regions were then imported into ClinProTools 3.0 (Bruker Daltonik) where they underwent smoothing, baseline subtraction, mass spectral alignment and normalization. Mann–Whitney non-parametric tests were performed using GraphPad Prism software.

## Figures and Tables

**Table 1:** MALDI-MSI *m*/*z* values with specific localization in the intima or media layer are shown (left column): x^p^ means specifically located in the calcified region of the intima layer. Comparison between healthy and atherosclerotic tissues is also included (right column):↑increased in atherosclerosis;↓decreased in atherosclerosis; *P*: pathologic (atherosclerotic) tissue; *C*: control (healthy) tissue. Bold numbers show statistical significance (*p* Value <0.05, Mann–Whitney test). Identification was performed by FT-ICR measurements, MaTisse database, MSiMass list database and literature [12,13].

	**Arterial localization**			**Atherosclerosis**			**Molecule**
***m*****/*****z***	**Media**	**Intima**	***p*****-Value**	**Trend**	**Fold change (*****P*****/*****C*****)**	***p*****-Value**	
**Proteins**							
**3011**		x	**0.0108**	↑	1.67	**0.0022**	SEL1L, IQGAP1, GANAB, NCSTN, UGDH, CYBA, YWHAG, MIF, EIF2S3, SYNM, ITGA5, NDUFS7, COL12A1, VASN, EEF1A1, MYBPC1, HBA1-2, ENO1, UBA1, CA3, MUC5B
**3553**	x		**0.0022**	↓	0.64	**0.0152**	NSF, PSMC4, ACTB, MYL2, PKM2, HSPD1
**3569**	x		**0.0022**	↓	0.67	**0.0303**	DHRS7, ACTB, MYL2, PKM2, ERP44, S100A6
**4597**	x		**0.0022**	↓	0.92	0.4589	–
**4614**	x		**0.0022**	↓	0.93	0.6494	HBB
**4762**		x	**0.0303**	↑	3.00	**0.0022**	TMSB4X
**4778**		x	**0.0303**	↑	2.07	**0.0022**	–
**5620**	x		**0.0022**	↓	0.58	**0.0087**	–
**6182**	x		**0.0022**	↓	0.49	**0.0022**	–
**6199**	x		**0.0022**	↓	0.57	**0.0152**	–

**Lipids**							
**255**		x	**0.0152**	↑	4.98	**0.0022**	SFA
**518**		x	**0.0022**	↑	8.74	**0.0022**	Lysolipids
**520**		x	**0.0260**	↑	4.58	**0.0260**	Lysolipids
**522**		x	**0.0022**	↑	5.64	**0.0022**	LPC (0:0/18:1), lysolipids
**535**		x^P^	**0.0381**	↑	4.21	**0.0381**	–
**536**		x^P^	0.3524	↑	1.42	0.1714	–
**568**		x^P^	0.1714	↑	3.57	0.0667	–
**675**		x^P^	0.0667	↑	6.84	**0.0190**	PA
**676**		x^P^	0.1143	↑	4.61	**0.0381**	PA+PG
**691**		x^P^	0.0667	↑	4.43	**0.0381**	SM+PA+PE−Cer
**722**		x^P^	0.1143	↑	4.76	0.1143	PC+PE
**800**		x	**0.0022**	↑	3.74	**0.0022**	SM
**864**		x	**0.0087**	↑	9.77	**0.0022**	PG
**865**		x	**0.0087**	↑	6.54	**0.0022**	PI
**866**		x	**0.0260**	↑	1.03	**0.0022**	PC
**891**		x	0.0931	↑	6.52	**0.0022**	Glc−GP+PI
**893**		x	**0.0433**	↑	6.18	**0.0411**	PS
**895**		x^P^	0.3874	↑	1.51	0.1320	TG
